# Bicolored White-toothed Shrews as Reservoir for Borna Disease Virus, Bavaria, Germany

**DOI:** 10.3201/eid1912.131076

**Published:** 2013-12

**Authors:** Manon Bourg, Sibylle Herzog, Jorge A. Encarnação, Daniel Nobach, Hildburg Lange-Herbst, Markus Eickmann, Christiane Herden

**Affiliations:** Justus-Liebig-University, Giessen, Germany (M. Bourg, S. Herzog, J.A. Encarnação, D. Nobach, H. Lange-Herbst, C. Herden);; Philipps University, Marburg, Germany (M. Eickmann)

**Keywords:** Borna disease, Borna disease virus, viruses, bicolored white-toothed shrews, Crocidura leucodon, reservoir, sequence analysis, RT-PCR, nested PCR, immunohistochemical analysis, indirect immunofluorescence assay, Bavaria, Germany

**To the Editor:** Borna disease (BD) is a fatal neurologic disorder in horses and sheep. The etiologic agent, Borna disease virus (BDV), belongs to the order Mononegavirales, which is composed of many reservoir-bound, highly pathogenic, and zoonotic viruses.

To investigate whether small mammals, especially bicolored white-toothed shrews (*Crocidura leucodon*), which act as BDV reservoirs in Switzerland (*1**,**2*), harbor BDV in disease-endemic areas in Bavaria, Germany, we screened 120 small mammals (53 from the family Cricetidae, 41 from the family Muridae, and 26 from the family Soricidae) (Table). We also determined whether BDV infections in small mammals might have different disease courses and whether shrew-to-horse virus transmission occurs.

**Table:** Small mammals from 7 stables tested for Borna disease virus infection, Bavaria, Germany, 1997–2012*

Stable, species	Common name	No. tested	No. positive for antibodies against BDV	No. positive for BDV RNA by RT-PCR
A				
* Sorex araneus*	Common shrew	2	0/2	0/2
* Mus musculus*	House mouse	17	2/13†	0/17
* Apodemus sylvaticus*	Wood mouse	1	0/1	0/1
*Microtus* sp.	Vole	1	0/1	0/1
B				
* Crocidura leucodon*	Bicolored white-toothed shrew	1	1/1	1/1
* Mus musculus*	House mouse	2	0/2	0/2
C				
* Micromys minutus*	Harvest mouse	1	1/1	0/1
* Mus musculus*	House mouse	3	0/2†	0/3
* Myodes glareolus*	Bank vole	1	0/1	0/1
*Microtus* sp.	Vole	1	0/1	0/1
D				
*Microtus* sp.	Vole	2	0/2	0/2
E				
* Crocidura leucodon*	Bicolored white-toothed shrew	19	3/13†	1/19
* Crocidura russula*	Greater white-toothed shrew	1	0/1	0/1
* Sorex araneus*	Common shrew	3	1/3	0/3
* Micromys minutus*	Harvest mouse	1	0/1†	0/1
* Mus musculus*	House mouse	6	0/6	0/6
* Apodemus sylvaticus*	Wood mouse	5	0/5	0/5
* Apodemus flavicollis*	Yellow-necked mouse	1	0/1	0/1
* Arvicola terrestris*	European water vole	1	0/1	0/1
*Microtus* sp.	Vole	34	0/32†	0/34
F				
* Apodemus sylvaticus*	Wood mouse	1	0/1	0/1
* Myodes glareolus*	Bank vole	1	0/1	0/1
* Arvicola terrestris*	European water vole	7	0/6†	0/7
*Microtus* sp.	Vole	2	0/1†	0/1†
G				
* Apodemus sylvaticus*	Wood mouse	3	0/3	0/3
* Apodemus flavicollis*	Yellow-necked mouse	1	0/1	0/1
* Arvicola terrestris*	European water vole	1	0/1	0/1
*Microtus* sp.	Vole	1	0/1	0/1

*BDV, Borna disease virus; RT-PCR, reverse transcription PCR. †No blood or brain samples were available.

The small mammals were captured during pest control efforts in stables in Upper Bavaria and Swabia that had a history of acute equine BD during 1997–2012. These stables also had a high probability for presence of *C. leucodon* shrews as documented by a recent distribution model (*3*). 

BDV-specific serum antibodies were identified by using an indirect immunofluorescence test and blood samples or thoracic or abdominal effusions as described (*4*). Antibodies against BDV were found in 8/105 specimens (Table) at serum dilutions ranging from 1:40 for *Mus musculus* mouse #1008, 1:80 for *M. musculus* mouse #1014, 1:2,560 for *C. leucodon* shrew #5063, 1:10,240 for *C. leucodon* shrew #2001, and 1:20,480 for *C. leucodon* shrew #5017.

Amplification of viral RNA was conducted by using real-time reverse transcription PCR (RT-PCR) (*5*) or nested RT-PCR (*6*) on 119/120 brain samples. In 2/4 BDV-seropositive *C. leucodon* shrews (#2001 and #5017) BDV RNA was amplified from the brain. The remaining 117 mice and insectivores were negative for BDV RNA, including 6/8 BDV-seropositive animals (Table).

Histologic and immunohistochemical (IHC) analyses for detection of BDV antigen were performed for small mammals that had antibodies against BDV or BDV RNA. In addition, histologic and IHC analyses were used to test 36/112 small mammals negative for BDV (by indirect immunofluorescence test and RT-PCR), including 15/16 *C. leucodon* shrews from 3 stables (B, C, and E), in which BDV-positive mammals were captured. None of the small mammals showed obvious gross or histologic lesions, even in the brain.

IHC analysis was performed by using monoclonal antibody Bo18 against BDV nucleoprotein (BDV-N) as described (*7*). The 2/2 *C. leucodon* shrews (#2001 and #5017) harboring viral RNA had BDV antigen in the central and peripheral nervous system (brain, spinal cord, spinal trigeminal ganglia, and peripheral nerves). Immunostaining of the skin showed evidence of BDV infection, mainly in epidermal keratinocytes and sebaceous glands, as well as in squamous epithelium and connective tissue of the esophagus. In shrew #5017, renal tubuli and glomeruli, as well as nuclei of bronchiolar epithelial cells, had BDV-N. No evidence for viral antigen was found in the other 42/44 small mammals tested.

In situ hybridization was performed by using established protocols (*8*). Viral genomic RNA and mRNA encoding for the BDV-N gene were found in the brain, spinal cord, ganglia, parotid gland, and sebaceous glands of the skin of 2 shrews positive for BDV by RT-PCR. Thus, BDV antigen and RNA were found in nervous tissue and peripheral organs of 2 *C. leucodon* shrews, as reported for shrews in Switzerland (*1**,**2*).

Viral dissemination into peripheral organs represents a prerequisite for successful viral excretion and transmission to other susceptible species. Simultaneous detection of viral genomic RNA and mRNA can indicate viral replication and transcription in peripheral organs. RNA from brains of the 2 BDV-positive *C. leucodon* shrews (#2001 and #5017) and from 1 horse that had BD and lived in the same stable as shrew #5017 was sequenced as described (*2*). Comparison of BDV sequence (GenBank accession no. KF275185) from *C. leucodon* shrew #5017 with sequence (GenBank accession no. KF275184) from the affected horse showed 100% identity in a 2,150-nt region (nt 17–2161 covering the N, X, and P genes and half of the M gene). Moreover, the BDV sequence showed 98% homology with those of the BDV isolates of the Baden-Wurttemberg and Bavaria II group (*9*).

The 2 BDV-positive shrews were trapped in April (#2001) and July (#5017) 2012 in different stables in the feeding area for hay (B for #2001) or in the storage area for feed (stable E for #5017), which probably indicates that this food was contaminated with BDV. Viral shedding in shrews might occur from skin, kidney, or gastrointestinal tract, which is similar to shedding by persistently infected, immunotolerant, neonatal Lewis rats (*10*).

In conclusion, BDV RNA, viral antigen, and serum antibodies against BDV were detected in 2/20 *C. leucodon* shrews, indicating that this shrew is reservoir of BDV in Bavaria. Whether seropositivity without other evidence of BDV infection indicates different courses of infection in small mammals, as known for horses, is not known and warrants further investigation. The absolute homology of shrew and equine BDV suggests successful interspecies virus transmission. Our study provides reliable evidence that *C. leucodon* shrews acts as reservoirs for BDV in disease-endemic areas in Bavaria, Germany, argues for a general role of this shrew as a reservoir for mammalian bornaviruses.
